# RNN- and LSTM-Based Soft Sensors Transferability for an Industrial Process

**DOI:** 10.3390/s21030823

**Published:** 2021-01-26

**Authors:** Francesco Curreri, Luca Patanè, Maria Gabriella Xibilia

**Affiliations:** 1 Department of Mathematics and Computer Science, University of Palermo, 90123 Palermo, Italy; fcurreri@unime.it; 2 Department of Engineering, University of Messina, 98166 Messina, Italy; lpatane@unime.it

**Keywords:** soft sensors, dynamical models, system identification, sulfur recovery unit, RNN, LSTM, transfer learning

## Abstract

The design and application of Soft Sensors (SSs) in the process industry is a growing research field, which needs to mediate problems of model accuracy with data availability and computational complexity. Black-box machine learning (ML) methods are often used as an efficient tool to implement SSs. Many efforts are, however, required to properly select input variables, model class, model order and the needed hyperparameters. The aim of this work was to investigate the possibility to transfer the knowledge acquired in the design of a SS for a given process to a similar one. This has been approached as a transfer learning problem from a source to a target domain. The implementation of a transfer learning procedure allows to considerably reduce the computational time dedicated to the SS design procedure, leaving out many of the required phases. Two transfer learning methods have been proposed, evaluating their suitability to design SSs based on nonlinear dynamical models. Recurrent neural structures have been used to implement the SSs. In detail, recurrent neural networks and long short-term memory architectures have been compared in regard to their transferability. An industrial case of study has been considered, to evaluate the performance of the proposed procedures and the best compromise between SS performance and computational effort in transferring the model. The problem of labeled data scarcity in the target domain has been also discussed. The obtained results demonstrate the suitability of the proposed transfer learning methods in the design of nonlinear dynamical models for industrial systems.

## 1. Introduction

Soft Sensors (SSs) are mathematical models of industrial processes able to estimate hard-to-measure variables (i.e., quality variables) by exploiting their dependence on easy-to-measure variables (i.e., quantity variables) [1,2]. SSs are widely adopted in industrial processes to improve process monitoring and control. Real-time quality variable estimation is necessary when quality variables are measured with large delays or require time-consuming laboratory analysis. In these cases, the design of an SS allows for increasing the performance of feedback control strategies. SSs are widely diffused in process industries, such as refineries [3], chemical plants [4], cement kilns [5], power plants [6], pulp and paper mills [7], food processing [8], polymerization processes [9], or wastewater treatment systems [10].

SS implementation often requires the use of black-box nonlinear dynamical identification strategies, which uses data collected from the distributed control system [11] and stored in the historical database. To achieve this aim, machine learning (ML) techniques are mostly used, ranging from Support Vector Regression [12], Partial Least Square [13], and classical multilayer perceptrons [1,14,15,16,17] to more recent deep architectures, such as deep belief networks [9,18,19,20], long short-term memory networks (LSTMs) [21,22], and stacked autoencoders [23,24,25,26]. Bayesian approaches [27], Gaussian Processes Regression [28], Extreme Learning Machines [29], and adaptive methods, [30,31,32] are also used.

Data-driven SS design can be summarized in the following steps, which are typical of the system identification procedure [33]:

- data acquisition, selection, and pre-processing;
- model class selection;
- model order selection;
- model identification; and
- model validation.

The design phase involves a lot of open problems and time-consuming tasks [34,35]. Among these we can mention: input-variable choice, model-class selection (e.g., linear/nonlinear, static/dynamic, time-variant/invariant), model-order design, model-structure, and hyperparameters selection. Another relevant problem is known as labeled data scarcity. In fact, conventional supervised learning algorithms, usually adopted in SS design, require the use of labeled data. While quantity variables are sampled with a fast rate, the corresponding quality variables are, in general, infrequently measured. This issue can be addressed by using semi-supervised learning, which exploits unlabeled data in an unsupervised training phase and labeled data in a supervised fine-tuning [19,36,37].

Since some industrial processes present a high nonlinearity and intrinsic dynamical dependencies between input and output variables, feed-forward artificial neural networks (ANNs) require the use of tapped delay lines (TDLs) for the I/O variables [1].

As an alternative, Recurrent Neural Networks (RNNs) can be used to catch temporal dynamics behaviors. In such networks, connections between hidden units are included between the previous and same level(s), making their output influenced by both the current and previous time instants. RNNs can therefore extract the sequential information available in the input data and can show better performance when modeling industrial processes.

To catch long-term dependencies among the variables, Long Short-Term Memory (LSTM) networks have been introduced. They contain memory cells that can store information for long periods of time during the training phase.

A common problem present in recurrent networks consists of a large number of hyperparameters to be optimized. Hyperparameters directly control the behavior of the training algorithm, and their correct setting strongly impacts the performance of the final model. Different hyperparameter optimization searching strategies are proposed in the literature, such as grid search, genetic algorithms, Bayesian Optimization, or Tree-structured Parzen estimators [38,39,40]. However, their optimization is an extremely computational and time-consuming task.

The outcome of the SS design process is a model tailored for the specific dataset adopted in the learning procedure, which should, therefore, cover all the working points of the plant. In general, the obtained model is not scalable without an adaptation to other processes. Developing SSs for a similar process requires, therefore, a new design procedure.

**Figure 1:**
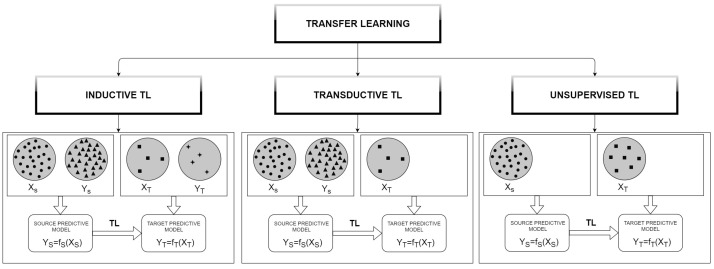
Basic flow of transfer learning. Source input ($X_{S}$), source output ($Y_{S}$), target input ($X_{T}$), and target output ($Y_{T}$).

As an effort to reduce the computational time required to design an SS for similar processes, model transferability plays a key role. Transfer learning (TL) focuses on storing the knowledge gained while learning a task from a source domain and utilizing it for a different but related problem, defined as the target domain [41]. TL techniques can be divided into three classes: inductive, transductive, and unsupervised transfer learning [42,43]. In the inductive TL, labeled data in the target domain are required to induce a predictive model (here named $f_{T}$) to be used in the target domain. In the transductive TL methods, no labeled data in the target domain are available, while they are available in the source domain. The unsupervised transfer learning focuses on solving unsupervised learning tasks in both the source and the target domains, such as clustering and dimensionality reduction. No labeled data are used. A scheme of the differences among the TL methods is reported in Figure 1.

TL methods are widely diffused in applications, such as classification, image processing, and natural language processing, as described in the next section. There actually exist few studies that investigate TL technique applications on industrial processes, both for SS design [44] and fault detection and diagnosis [45,46].

In our work, we focus on inductive transfer learning for SS design, when a limited number of labeled data is available in the target domain. Two different strategies are proposed. The first strategy, called the fine-tuned transferred model (FTTM), consists of performing only a fine-tuning of the network weights of the optimal model designed in the source domain, with the dataset belonging to the target domain. The second strategy, called the transferred hyperparameters model (THM), is based on adopting only the optimal hyperparameters identified in the source domain to train the SS in the target domain, starting from random initial weights. RNN- and LSTM-based SSs are considered and compared in regard to transferability properties. The use of the proposed techniques allows, at the same time, to reduce the time needed to design a SS for a similar process and to cope with the problem of labeled data scarcity.

The transferability of SSs between two similar industrial processes is considered in our work. A Sulfur Recovery Unit (SRU) from a refinery located in Sicily (Italy) is considered as a case study. It is a highly nonlinear process with dynamic dependencies between input/output variables [47]. It consists of different lines that work in parallel. RNN and LSTM-based SSs have been designed for two lines of the process (i.e., SRU line 2 and SRU line 4). The transferability of models designed for SRU line 4 (i.e., the source domain) to SRU line 2 (i.e., the target domain) has been investigated.

The main contributions of this work are summarized as follows: (1) The TL methodologies are applied to SS design, which is a topic rarely considered in the TL research field. (2) The transferability of nonlinear dynamical models is considered. This aspect is relevant both in the field of SS design which, often, considers static models and in the TL applications. (3) Two dynamical neural models (i.e., RNN and LSTM) are designed and compared in regard to model accuracy and transferability. The trade-off between the performance and computational time required to transfer the SS from the source to the target domain is analyzed. (4) Two different TL approaches are presented and discussed. Both the adopted techniques have the advantage of avoiding the time-expensive procedure of hyperparameters selection for the target dataset. (5) A real-world industrial case study is considered. (6) The presented framework is successfully applied in two different scenarios, to outline the advantages in presence of labeled data scarcity in the target domain. To underline this aspect, analyses including datasets with similar size and datasets with a reduced amount of data for the target domain are reported.

The remainder of this paper is organized as follows: In Section 2, the state of the art on TL is reported; in Section 3, RNN and LSTM structures are explained in details, along with the SS implementation; in Section 4, the proposed TL methods are introduced; in Section 5, the case study is presented, and the numerical results of the TL procedures are reported and discussed. Conclusions are finally drawn in Section 6.

## 2. Related Works

Transfer learning is a relevant topic in the ML field, especially referring to deep learning strategies. Most of the theoretical results and applications belong to the area of classification, including fault detection applications. Only a few results are available in regard to SSs and regression estimation. In this section, some related works are briefly introduced. Examples of applications in different research areas, such as image classification [48,49], text classification [50], and biometrics [51], can be found in the literature. In Reference [52], a new multi-source deep transfer neural network algorithm, based on a convolutional neural network (CNN) and a multi-source TL technique, is proposed and evaluated on several classification benchmarks. A systematic analysis of computational intelligence-based TL techniques is reported in Reference [53]. Methods based on neural networks, Bayesian systems and fuzzy logic are described in the paper, along with applications in the field of language processing, computer vision, biology, finance, and business management. A structured description of the application fields and methodologies related to TL can be, also, found in References [41,42,53,54]. Some metrics suitable to evaluate the distance between domains are reported in Reference [43]. Applications on the industrial field, related to TL for process monitoring, are mostly dealing with fault detection tasks. In Reference [55], an application to a gearbox fault dataset, based on CNNs, is presented. In the paper, a CNN is trained on large datasets to learn hierarchical features from raw data. Both the architecture and weights of the pre-trained CNN are then transferred to a new task using a fine-tuning procedure. Different TL strategies have been compared to analyze feature transferability from the different levels of the structure. In Reference [56], a TL method for gas turbine fault diagnosis based on CNNs and support vector machines is proposed. The scarcity of information related to faults has been solved by applying a feature mapping method, reusing the internal layers of a CNN trained on the normal dataset. Another interesting approach of TL applied to CNNs is reported in Reference [57]. The proposed method addresses a qualitative tool condition monitoring problem, using computer vision, CNNs and TL approaches, to teach the machines the conformity of the component-producing tool. In Reference [58], a fault diagnosis method based on variational mode decomposition, multi-scale permutation entropy and feature-based TL is proposed. The methodology was applied to the vibration signal of wind turbines. In Reference [59], a linear discriminant analysis (LDA)–based on Deep transfer network is proposed for fault classification of chemical processes as the Tennessee Eastman benchmark and real hydrocracking processes. A maximum mean discrepancy based loss function is used to extract similar latent features and reduce the discrepancy of distributions between the source and target data. Domain-Adversarial Neural Networks are introduced as a domain adaptive TL technique in Reference [60], to implement transferable fault diagnosis. A fault diagnosis system is also developed in Reference [61] using an LSTM model, based on instance TL, to reduce the differences in the probability distributions of the source and the target domains. Other applications in the field of fault diagnosis are reported in References [62,63]. A few applications to SS design have been proposed in very recent works. In Reference [64], a domain adaptation, soft sensing framework for multi-grade chemical processes is discussed. A limited number of labeled samples is available for some operating grades. An adversarial transfer learning SS is proposed to reduce the data distribution discrepancy between different grades, therefore allowing for a supervised SS development. A similar approach, based on extreme learning machine, has been proposed in Reference [65] to develop an SS for a simulated continuous stirred tank reactor and an industrial polyethylene process. In Reference [25], a data-driven model based on deep dynamic features extracting and transferring methods are applied to build a virtual sensor for cement quality prediction. A large unlabeled dataset is used to extract nonlinear dynamic features, along with a limited labeled dataset. The features are then transferred to a regression model, called the eXtreme Gradient Boosting, for output prediction. A model updating strategy is also proposed to include online data samples. In Reference [66], an instance-based TL method is combined with a boosting decision tree. The procedure is adopted to estimate wind power generations and uses correlated zones of the source domain to realize an instance-based transfer learning.

## 3. Theory Fundamentals

In this section, a description of the RNN and LSTM architectures, used to identify the data-driven nonlinear dynamical models, is reported. Moreover, the details on the structures adopted in this work are provided.

### 3.1. Recurrent Neural Networks

RNNs [67] are widely used to capture the temporal dynamic behavior in time sequences [68]. RNNs can make use of past states and past information for the present state estimation, making them suitable for sequences processing, such as natural language, handwriting recognition, and speech recognition [69,70,71]. Such a property makes this type of networks able to identify dynamical models of industrial processes.

The intrinsic dynamic structure of the RNN allows it to avoid the regressor selection procedure needed when using static networks. It is also not necessary to feed past I/O samples into the input layer. RNNs have the same structure of multilayers perceptrons, with the difference that, in an RNN, neuron connections are included between the previous levels and in the same level, as well. This forms a directed graph along a temporal sequence since, at each instant, the nodes connected through a recurring connection receive inputs both from the current and previous state, based on the dependencies created in the network.

**Figure 2:**
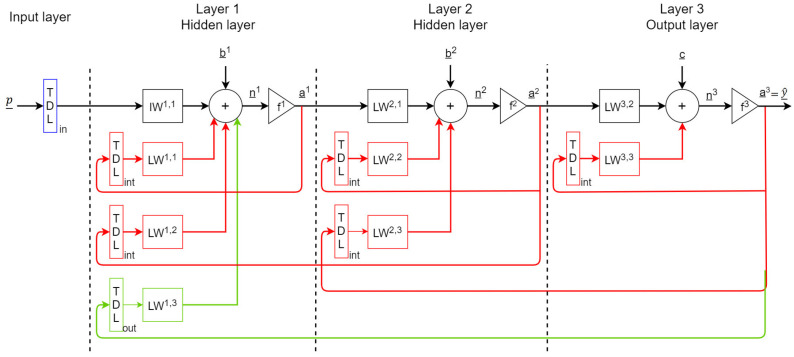
Block scheme of an Recurrent Neural Network (RNN) with two hidden layers with delays and recurrent connections.

The connections between the output of a layer and the input of the previous one are performed by applying a real-valued time-delay between them. Such delays are implemented with TDL blocks. Figure 2 shows an RNN with two hidden layers with input delays $TDL_{in}$, internal recurrent connections delays $TDL_{int}$ and output recurrent connections delays $TDL_{out}$.

Given a layer *ℓ*, its output $a^{\ell }\left(t\right)$ is given by

(1) $$a^{\ell }\left(t\right)=f^{\ell }\left(n^{\ell }\left(t\right)\right),$$

where $f^{\ell }$ is the activation function, particularly the hyperbolic tangent tanh in the hidden layers (i.e., $f^{1}$, $f^{2}$) and the linear function in the output layer (i.e., $f^{3}$). The input signals $n^{\ell }\left(t\right)$ are given by the following equations:

(2) $$\begin{array}{c} n^{1}\left(t\right)=IW^{1,1}\left[p\left(t\right);p\left(t-1\right);...p\left(t-TDL_{in}\right)\right]\\ +LW^{1,1}\left[a^{1}\left(t-1\right);...a^{1}\left(t-TDL_{int}\right)\right]\\ +LW^{1,2}\left[a^{2}\left(t-1\right);...a^{2}\left(t-TDL_{int}\right)\right]\\ +LW^{1,3}\left[a^{3}\left(t-1\right);...a^{3}\left(t-TDL_{out}\right)\right]+{\underline{b}}^{1}; \end{array}$$

(3) $$\begin{array}{c} n^{2}\left(t\right)=LW^{2,1}a^{1}\left(t\right)+LW^{2,2}\left[a^{2}\left(t-1\right);...a^{2}\left(t-TDL_{int}\right)\right]\\ +LW^{2,3}\left[a^{3}\left(t-1\right);...a^{3}\left(t-TDL_{int}\right)\right]+{\underline{b}}^{2}; \end{array}$$

(4) $$n^{3}\left(t\right)=LW^{2,2}a^{2}\left(t\right)+LW^{3,3}\left[a^{3}\left(t-1\right);...a^{3}\left(t-TDL_{int}\right)\right]+\underline{c}.$$

Matrix $IW^{1,1}$ contains the weights of the inputs; $LW^{1,1}$ is the internal feedback weight matrix in layer 1; $LW^{1,2}$ is the external feedback weight matrix in layer 2; $LW^{1,3}$ is the external feedback weight matrix in layer 3 (i.e., the output layer). $LW^{2,1}$ is the layer weight matrix between layer 2 and layer 1; $LW^{1,2}$ is the internal feedback weight matrix in layer 2. $LW^{3,2}$ is the matrix of the weights of the output layer; $LW^{3,3}$ is the matrix of the internal feedback weight in the output layer. The vectors ${\underline{b}}^{1}$, ${\underline{b}}^{2}$, and $\underline{c}$ contain the bias values for layers 1 and 2 and the output layer, respectively. In particular, the vector $\left[p\left(t\right);p\left(t-1\right);...p\left(t-TDL_{in}\right)\right]$ is built from the input vector at time *t* and the consecutive tapped input delays; the vectors $\left[a^{\ell }\left(t-1\right);...a^{\ell }\left(t-TDL_{int}\right)\right]$ are built from the layer *ℓ* output delayed in the value of $TDL_{int}$ (or $TDL_{out}$) to itself.

The RNNs are here trained both with the Levenberg-Marquardt (LM) algorithm [72] and the Broyden-Fletcher-Goldfarb-Shanno (BFGS) algorithm [73].

However, standard RNNs have difficulties in learning long-term dependencies, because they are easily affected by the vanishing or exploding gradient problem [74]. This issue occurs when the gradient becomes vanishingly small, at the point of preventing the weights from changing value, or vice-versa, when it increases exponentially, making the derivatives diverge.

### 3.2. Long Short-Term Memory Network

**Figure 3:**
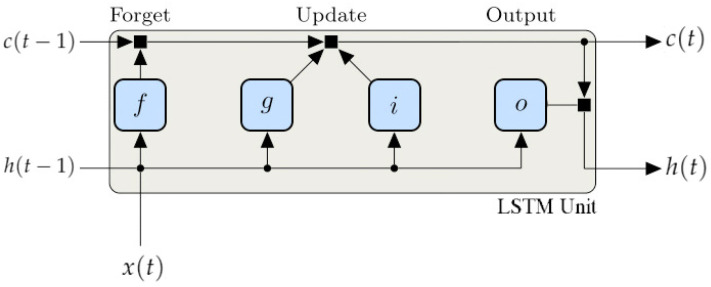
Working diagram of a long short-term memory network (LSTM) unit, showing how the gates forget, update, and output both cell and hidden states.

LSTM networks have been introduced as a variant to standard RNNs to deal with such issues [75]. Basic hidden units in RNNs are replaced with LSTM units, making the network handle the vanishing and exploding gradient problem when learning long-term dependencies [76]. LSTM units consist of memory cells and three nonlinear gates that selectively retain current information that is relevant and forget past information that is not relevant. This type of network is mostly used in language modeling, time series prediction, speech recognition and video analysis [77,78,79,80]. An LSTM unit is shown in Figure 3.

Given a time instant *t*, the state of the unit consists of the hidden (or output) state h(t), which contains the output for that time instant, and the cell state c(t), which contains information learned from previous time instants. They are computed using h(t−1) and c(t−1) from the previous time step. At each time step, c(t) is updated by adding or removing information using gates. The blocks that form the LSTM unit and control the next state are the following:

- Forget gate (*f*), that controls the level of cell state reset;
- Cell candidate (*g*), that adds information to cell state;
- Input gate (*i*), that controls the level of cell state update;
- Output gate (*o*), that controls the level of cell state added to the hidden state.

The following are the learnable parameters of an LSTM layer:

1. Input weights: $W^{i},W^{f},W^{g},W^{o};$
2. Recurrent weights: $R^{i},R^{f},R^{g},R^{o};$
3. Biases: $b^{i},b^{f},b^{g},b^{o}.$

The states of the blocks of the LSTM unit at the time instant *t* can be written as:

(5) $$\begin{array}{c} f\left(t\right)=\sigma_{g}\left[W^{f}X\left(t\right)+R^{f}h\left(t-1\right)+b^{f}\right];\\ g\left(t\right)=\sigma_{c}\left[W^{g}X\left(t\right)+R^{g}h\left(t-1\right)+b^{g}\right];\\ i\left(t\right)=\sigma_{g}\left[W^{i}X\left(t\right)+R^{i}h\left(t-1\right)+b^{i}\right];\\ o\left(t\right)=\sigma_{g}\left[W^{o}X\left(t\right)+R^{o}h\left(t-1\right)+b^{o}\right]; \end{array}$$

where $\sigma_{g}$ denotes the sigmoid function, and $\sigma_{c}$ the hyperbolic tangent function tanh.

The cell state c(t) and the output state h(t) at each time instant are updated as:

(6) $$\begin{array}{c} c(t)=f(t)⊙c(t-1)+i(t)⊙g(t);\\ h\left(t\right)=o\left(t\right)⊙\sigma_{c}\left(c\left(t\right)\right), \end{array}$$

where ⊙ denotes the Hadamard product, the pointwise multiplication operator for two vectors.

Given the learning rate α>0, the standard SGD algorithm updates the network parameters θ (weights and biases) to minimize the loss function E(θ) by taking small steps at each iteration *k* in the direction of the negative gradient of the loss as follows:

(7) $$\theta_{k+1}=\theta_{k}-\alpha \nabla E\left(\theta_{k}\right).$$

SGDM adds momentum to reduce the possible oscillation along the path of steepest descent towards the optimum.

(8) $$\theta_{k+1}=\theta_{k}-\alpha \nabla E\left(\theta_{k}\right)+\gamma \left(\theta_{k}-\theta_{k-1}\right).$$

The term γ determines the contribution of the previous gradient step to the current iteration. Even though Adam optimizer [81] is more computationally efficient, SGDM showed better performances in our applications.

### 3.3. Model Description

In this section, some notations and technical details that will be used in the following of the paper are reported.

Let us denote the model implemented by a generic network (i.e., RNN and LSTM) y=f(w,h), where w is a vector containing all the network weights and biases, and h contains all the model hyperparameters, thus describing the network structure. In the case of RNN, the hyperparameters are detailed as follows:

1. Number of input delays;
2. Number of internal delays;
3. Number of output delays;
4. Number of hidden layers;
5. Number of neurons for each hidden layer;

The LSTM model training involves the optimization of the following hyperparameters:

1. Number of hidden units in the LSTM layer;
2. Number of hidden neurons in the fully connected layer;
3. Dropout probability value.

The Dropout, a technique to prevent over-fitting in deep neural networks, has been applied. It consists of randomly disconnecting some neurons by a certain percentage during the training, by setting their outgoing edge to 0 at each epoch. This way, at each update during the training phase, each neuron has a probability to be dropped out and miss the training [82]. Among the available hyperparameters searching strategies, a grid search approach was preferred in both the RNN and LSTM cases.

Model performances were evaluated through CC, MAE, and RMSE between the actual output and the predicted output over test data as follows:

(9) $$CC=\frac{cov(Y,\hat{Y})}{\sigma_{Y}\sigma_{\hat{Y}}},$$

(10) $$MAE=\frac{\sum_{i=1}^{N}\left|y_{i}-\hat{y_{i}}\right|}{N},$$

(11) $$RMSE=\sqrt{\frac{\sum_{i=1}^{N}{\left(y_{i}-\hat{y_{i}}\right)}^{2}}{N}},$$

where cov(·) is the covariance, σ the standard deviation, *N* is the number of samples, $y_{i}$ and $\hat{y_{i}}$ are the actual and the predicted output samples, and *Y* and $\hat{Y}$ are the corresponding vectors. To select the optimal SS and to compare the different methodologies here reported, the CC is considered.

The experiments were performed on a laptop with an Intel i5 @2.4GHz CPU and 8 GB RAM. The software environment is Mathworks MATLAB 2020a on Windows 10 Pro 64-bit.

## 4. Methodology

In this section, the proposed TL methods are described. The methods are applied in the hypothesis that a limited number of labeled data is available in the target domain. Therefore, they can be classified as inductive TL methods. Two different strategies are proposed. The FTTM consists of performing only a fine-tuning of the optimal SS structure designed in the source domain, using the target domain dataset. The THM adopts only the optimal hyperparameters identified for the source domain to train the SS for the target domain.

### 4.1. Fine-Tuned Transferred Model

The FTTM for TL is a well-known procedure often adopted, particularly in deep neural structures, for classification tasks. A typical example is the adaptation of complex CNN classifiers to different tasks, reusing a feature extraction layer, pretrained on a different domain, while adapting the final classification layer to the new domain [55]. Concerning SS design, the objective is to transfer the information related to the dynamical equation structure, which represents, in a black-box approach, the model of the considered system. When a similar process needs to be identified, the TL procedure allows to leave out a number of time-consuming phases. In particular, input-variable choice, model-class selection, model-order design, model-structure, and hyperparameters selection are transferred from the source to the target domain. As stated in the previous section, this information is contained in the vectors $w_{S}$ and $h_{S}$, where the subscript *S* states for the source domain. They have been obtained identifying the model in the source domain (i.e., $x_{S}$, $y_{S}$) with a complex optimization phase. In detail, both the expert knowledge, the correlation analysis and a trial and error procedure have been used to select the model inputs. A grid search has been applied to select the optimal hyperparameters on the basis of a statistical analysis of the obtained performance.

In the FTTM approach, the weight matrix, previously trained for the source domain, is, therefore, used as an initial state for the fine-tuning and only a very limited number of training cycles is performed on the target domain dataset (i.e., $x_{T}$, $y_{T}$). The FTTM procedure is briefly reported in Algorithm 1.
**Algorithm 1:** FTTM Algorithm.  Load $f_{S}\left(w_{S},h_{S}\right)$;  Load $x_{T}$, $y_{T}$;  Fine-tune $f_{S}\left(w_{S},h_{S}\right)$ with $x_{T}$, $y_{T}$;  Test $f_{T}\left(w_{T},h_{S}\right)$  Compute MAE, RMSE, CC;


### 4.2. Transferred Hyperparameters

This second procedure does not use the knowledge contained at the level of the network weights. In fact, the network structure optimized to design the SS in the source domain is trained with the dataset of the target domain, starting from random initial weights. The only information transferred from the source domain is therefore contained in the hyperparameter vector (i.e., $h_{s}$) which contains information on the model order and structure.

The THM requires full training of the network and, therefore, a greater computational effort with respect to FTTM. In addition, it has to be noticed that, to avoid overfitting phenomena, an adequate number of labeled training data in the target domain is required. This aspect makes this procedure less useful in the case of labeled data scarcity. The THM procedure is briefly reported in Algorithm 2.
**Algorithm 2:** THM Algorithm.  Load $h_{s}$;  Load $x_{T}$, $y_{T}$;  Inizialize $f_{T}\left(w,h_{S}\right)$  Train $f_{S}\left(w,h_{S}\right)$ with $x_{T}$, $y_{T}$;  Test $f_{T}\left(w_{T},h_{S}\right)$  Compute MAE, RMSE, CC;


## 5. Simulation Results

In this section, the proposed procedure is applied to an industrial case study. Both the TL approaches (i.e., FTTM and THM) and the proposed neural approaches (i.e., RNN and LSTM) are applied. The obtained performances are compared in regard to the prediction accuracy and the computational complexity required to transfer the SS to a different process.

### 5.1. Case of Study: The Sulfur Recovery Unit

**Figure 4:**
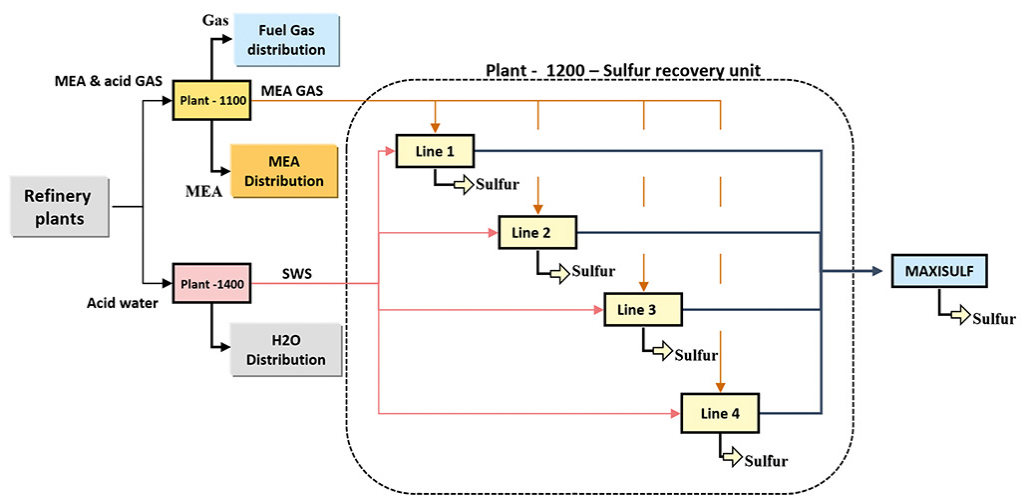
The industrial Sulfur Recovery Unit (SRU) taken into account contains four parallel processing lines.

The considered process is an SRU desulfuring system of a refinery located in Sicily, Italy, and detailed in Reference [47]. SRUs are employed in refineries to recover elemental sulfur from gaseous hydrogen sulfide ($H_{2}S$), usually contained in by-product gases derived from refining crude oil and other industrial processes. This is done through a gas desulfurizing process. The process is of fundamental importance, being $H_{2}S$ a dangerous environmental pollutant. The SRU consists of four parallel sub-units, called sulfur lines, as depicted in Figure 4.

**Figure 5:**
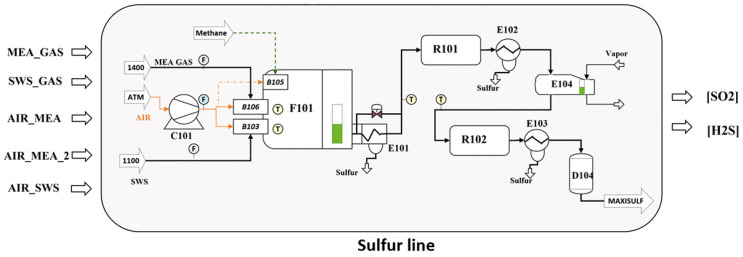
Simplified working scheme of an SRU line.

Each SRU line takes as inputs two kinds of acid gases: MEA gas rich in $H_{2}S$, and SWS (Sour Water Stripping) gas rich in $H_{2}S$ and ammonia ($N_{H}3$). They are burnt in reactors in two separate chambers along with a suitable airflow supply, to regulate the combustion. The final gas stream contains residuals of $H_{2}S$ and sulfur dioxide ($SO_{2}$). In Figure 5, a simplified working scheme of an SRU line is shown.

The sensors used to measure the concentrations of both $H_{2}S$ and $SO_{2}$ in the tail gas are often taken off for maintenance, making an SS needed to estimate their concentrations.

The input and output variables used in the models are listed in Table 1.

**Figure 6:**
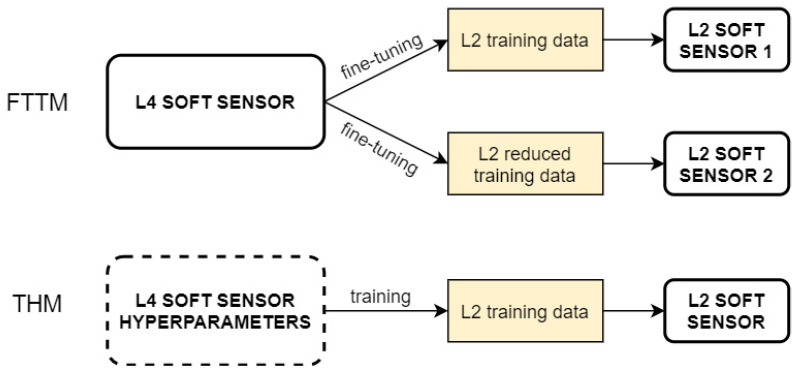
Transfer learning (TL) strategies applied to the SRU lines.

The available datasets consist of 14,401 samples from SRU line 2 and 10,081 samples from SRU line 4. Samples were collected with a sampling period of one minute. Outliers were manually removed by interpolation and the datasets were normalized with z-score normalization. The first 80% of the data are used for training and validation, and the remaining 20% for the test phase. SRU line 4 is considered as the source domain, whereas SRU line 2 represents the target domain. To simulate the scenario of labeled data scarcity, only 20% of the target domain training data was used. Figure 6 describes the simulations reported in the following to validate and compare the TL-approaches’ behavior.

### 5.2. Design of the Optimal SSs

The first SS in the source domain is based on RNN. Hyperparameter optimization is performed through a grid search. For each combination, five models are considered to apply a statistical analysis. The final evaluation is performed on the test dataset. TDLs are designed containing up to five time steps, with 125 possible delays combinations. RNN structures, with a maximum of three hidden layers, are considered, adopting the same number of neurons for each layer, in the range [2–5]. For sake of comparison, an SS has also been designed from scratch for the target domain, using the same ranges for the hyperparameters.

**Figure 7:**
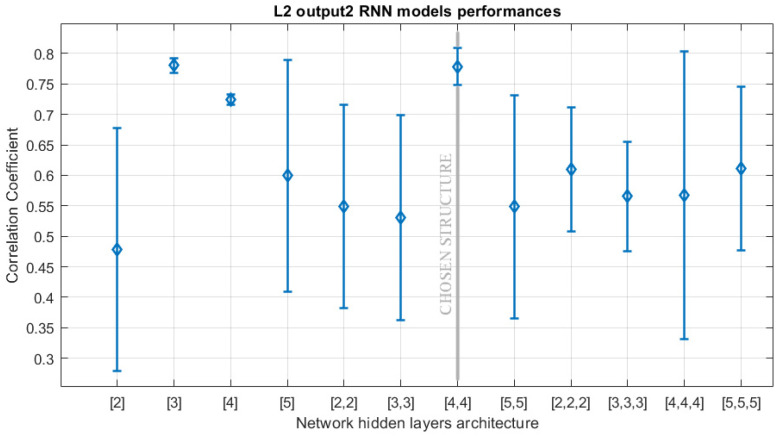
Statistical distribution of the CC on test data for different RNN network architectures. The output 2 for SRU line 2 is considered. The circles indicate the mean value, whereas the bars represent the standard deviation. The final selected structure is highlighted.

For each SS, the searching strategy required about 100 h of computational time. The training for the SS design has been performed with the BFGS algorithm. Figure 7 shows the statistical distribution of the CC of the output 2 of SRU line 2, between the predicted output and the measured one, sf function of the number of layers, and neurons in the network topology. The final RNN model hyperparameters for the optimized SS in the source and target domains are reported in Table 2. The final model for the source domain (i.e., SRU line 4) has been trained in 18 min and required 300 training epochs. The final model for target domain (i.e., SRU line 2) was trained in about 29 min, with the same number of epochs.

**Figure 8:**
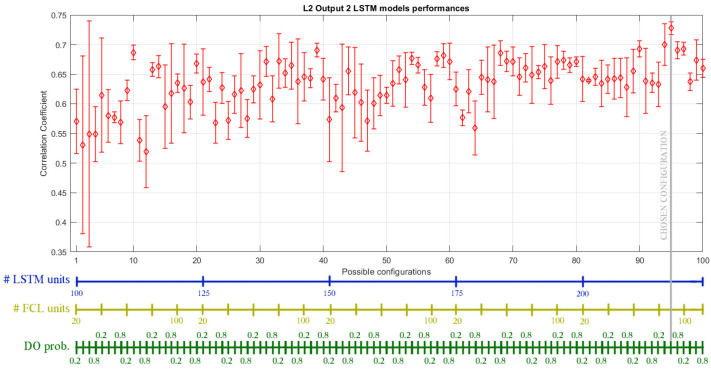
Statistical distribution of the CC on test data for different LSTM network architectures for the design of the SS modeling the output 2 of SRU line 2. The circles indicate the mean value, whereas the bars represent the standard deviation. Hyperparameter values are reported in the three colored bars below, and the chosen combination is highlighted.

The second set of SSs, based on the LSTM approach, has been designed by using a grid search in the following hyperparameters ranges: the number of hidden units of the LSTM layer is varied between 100 and 200, with a step size of 25; the number of hidden neurons in the fully connected layer between 20 and 100, with a step size of 20; the dropout probability between 0.5 and 0.8, with a step size of 0.1. For each of the 100 possible combinations, five networks have been generated and trained for 150 epochs with the SGDM algorithm. The simulations took about 90 h for each SS. Figure 8 shows the statistical distribution of the CC for output 2 of the SRU line 2. The final selected optimal structures are reported in Table 3.

The final models took 150 training epochs for SRU line 2, executed in 18 min, and 200 epochs for SRU line 4, in 16 min.

**Figure 9:**
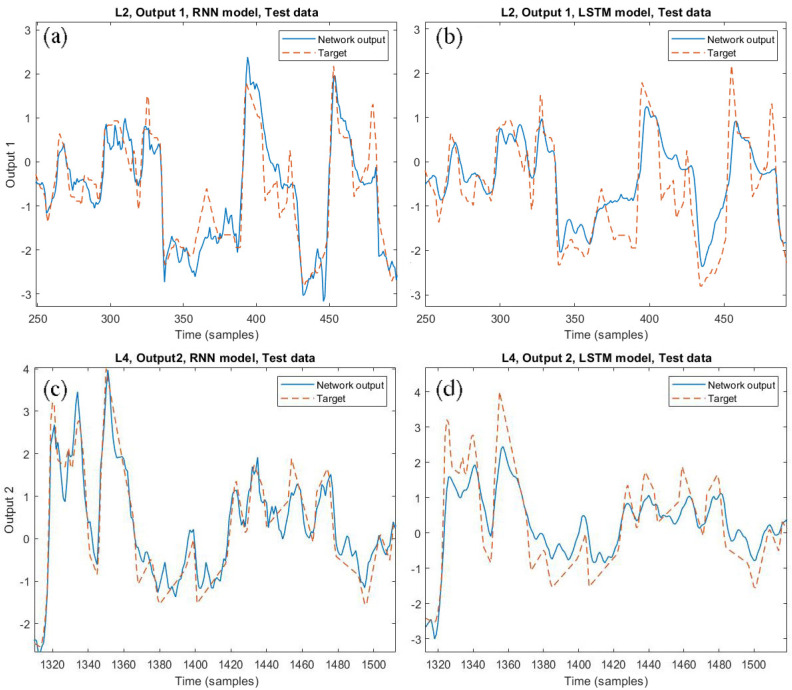
Network outputs and measured ones comparison for: the SRU line 2 output 1 with the RNN (**a**) and the LSTM model (**b**); the SRU line 4 output 2 with the RNN (**c**) and LSTM model (**d**).

The performance for both RNN and LSTM-based SSs are reported in Table 4 (SRU line 2) and Table 5 (SRU line 4). Figure 9 shows the network outputs and the measured one for output 1 of the SRU line 2 and output 2 of the SRU line 4 for both the RNN and the LSTM models.

### 5.3. Transferred Models

To evaluate the performance of the proposed TL methods, the SS optimized for the SRU line 4, called in the following SSL4, has been transferred to the SRU line 2. As a first step, simulations have been performed by evaluating the SSL4 on the target dataset without any modification. The results of these simulations are reported in Table 6 (CC) and in Table 7 (MAE and RMSE). It is evident that, for the SSs designed with both RNN and LSTM, there is a relevant degradation of the performance when transferring the model without modifications.

#### 5.3.1. Fine-Tuned Transferred Models

In this section, the results of the FFTM are reported. As described in Algorithm 1, the SSL4 is fine-tuned on the target domain. In the case of RNN model, the fine-tuning was performed with the LM algorithm and requested 6 epochs (45 s) before incurring into overfitting. Although the requested epochs are very few, the improvement obtained, as reported in Table 8, is significant. In fact, the previously computed performance degradation, with respect to the optimized SS for SRU line 2, is halved in the case of the RNN model (from −22.62% to −10.71% for output 1, and from −16.6% to −7.14% for output 2).

In the case of the LSTM model, the FTTM requested 75 epochs (7 min).The performances after the TL are listed in Table 8, along with the percentage degradation with respect to the optimized models. Errors are reported in Table 9. In this case, the number of requested epochs is larger than the RNN solution. The improvement obtained, as reported in Table 8 is from −37.5% to 0% for output 1, and from −11.11% to 0% for output 2. The LSTM structure was, therefore, able to reach, after the FTTM transferring, the same CC of the optimized SS. It can be noticed that the TL procedure required about 7 min, while the complete optimization phase for the corresponding SS required more than 100 h. The obtained results demonstrate that the FTTM procedure is largely effective for both architectures. In particular, for the LSTM model, we closed the gap between the optimized model performance and the transferred solution. The best prediction performance was, however, obtained with the RNN structure, as previously reported in Table 4.

**Figure 10:**
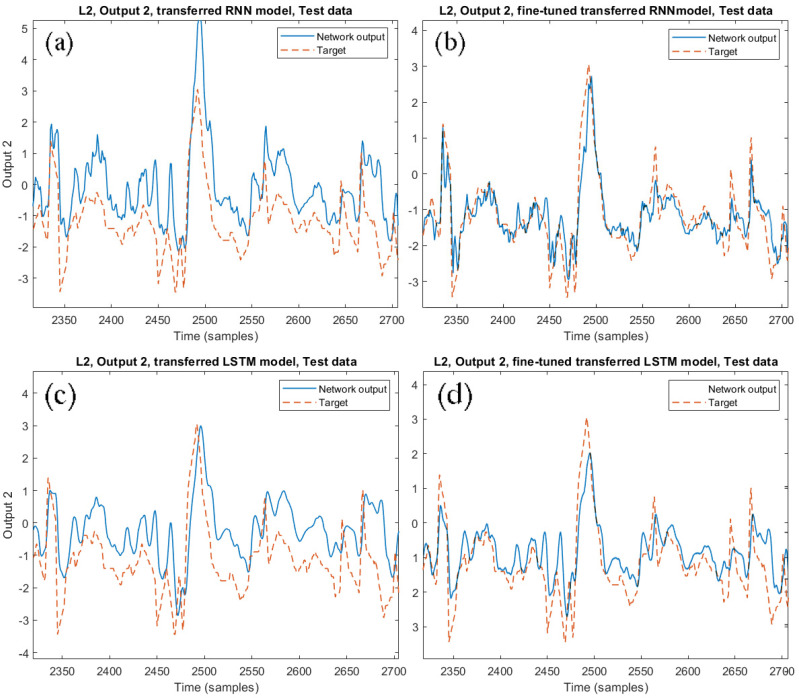
Comparison between the transferred RNN models on the SRU line 2 before (**a**) and after (**b**) the re-tuning. The same comparison is shown for the LSTM model (**c**,**d**).

Figure 10 shows the estimation of output 2 from the SRU line 2 obtained with the RNN model before (a) and after (b) the TL. The same comparison is reported for the LSTM model in Figure 10c,d. The results demonstrate that, even without fine-tuning, the SSL4 is able to catch the system dynamics also for the SRU line 2. However, a relevant error is present, which is largely reduced through the FTTM procedure.

As stated in the introduction, a key aspect of the TL is the possibility to handle labeled data scarcity. To investigate the transferability in such case, we considered a reduced version of the SRU line 2 dataset with only 2304 samples extracted from the original 11,520 training samples. Following the FTTM procedure, also in this case, the LSTM-based SS obtained better performance as shown in Table 10. A small degradation of the CC, compared with the optimized model trained with the entire dataset, is obtained. Errors are reported in Table 11.

#### 5.3.2. Transferred Hyperparameters Models

**Figure 11:**
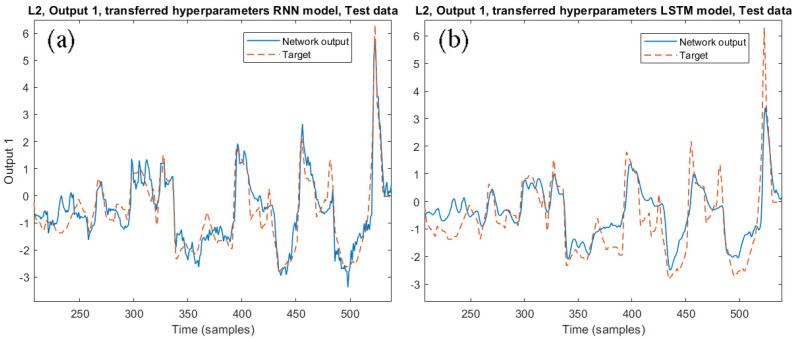
Comparison between the transferred models on the SRU line 2 through the THM technique: (**a**) RNN and (**b**) LSTM models.

As reported in Algorithm 2, the THM consists of maintaining only the optimal hyperparameters from the source model, performing a complete learning phase with the target dataset. The RNN model required 200 training epochs (16 min), using the BFGS algorithm. The LSTM model required 150 training epochs (16 min).The obtained performances are reported in Table 12 in term of CC, including the percentage degradation with respect to the optimized model performance. The corresponding errors are reported in Table 13. Figure 11 shows the network output 1 for the transferred SS. In addition, in this case, the reduced-size dataset has been used to apply the THM procedure, both with RNN and LSTM models. The results obtained are not satisfactory, confirming that the FTTM is more suitable to realize model transferring.

## 6. Conclusions

The proposed work investigates the problem of dynamical model transferability in developing SSs for industrial applications. Two different model transferability solutions were investigated. The first strategy (i.e., the fine-tuned transferred model) consisted in adopting the SS designed for the source domain, after a fine-tuning of the network weights on the target domain. The second strategy (i.e., the transferred hyperparameters model) is based on adopting the optimal hyperparameters identified for the source dataset to train a new SS on the target dataset. Both techniques have been implemented by using, as SS structures, two dynamical neural networks: an RNN and an LSTM. The RNN-based SS showed better performance than the LSTM network in developing the optimal model, with a comparable computational effort. The obtained results showed that the FTTM reached the best performance in terms of the correlation coefficient between the estimated SS outputs and the measured ones. In regard to the comparison between the two different ML approaches, the LSTM showed a greater capability of maintaining, after the transfer process, similar performance, when compared with the full optimization procedure. Another relevant achievement of the proposed procedures is the possibility to cope with the problem of labeled data scarcity. In the case of a limited labeled dataset, the LSTM showed the best transferring capabilities, with respect to the RNN-based model with the FTTM. The results obtained with the hyperparameter transferring are instead not satisfactory, confirming that the FTTM is more suitable as a TL procedure. The proposed application has shown the suitability of TL procedures in the field of SS for industrial processes, where dynamical nonlinear models are of interest. This is a relevant achievement in the SS research field, allowing to greatly reduce the computational complexity of the SS design. In detail, the use of TL allowed to leave out the time-consuming phases of model-class and order selection and hyperparameters optimization. Another relevant aspect of the proposed procedures, with respect to those reported in the literature, is their simplicity. The FTTM and the HTM algorithms can, in fact, be implemented directly from the industrial technicians, without the help of a system identification or ML expert. A set of labeled data in the target domain is, however, required. The proposed TL procedures have the characteristic to preserve the knowledge on the structure of the model dynamics from the source process to the target one. If the sensors available on the target process are different from those installed in the source one, this characteristic should still guarantee good performance, if the measured quantity variables are strictly related in the two domains. In the proposed application, the suitability of using a TL approach was assured by using the knowledge of the experts, who assessed the similarity between the two considered processes. In more general cases, this is not always easy to understand. A limitation of the proposed approaches consists, therefore, in the lack of a procedure able to assess the possibility of successfully applying TL to a given process, by looking only at the available datasets. Further research will be devoted to the introduction of proper metrics able to quantify the distribution distance between the source and target domains. This preliminary analysis should be able to guarantee the possibility of applying TL and, eventually, estimate the expected performance.

## Figures and Tables

**Table 1:** Input and output variables adopted in the SRU line models.

Variable	Description
$x_{1}$	gas flow in the MEA chamber
$x_{2}$	air flow in the MEA chamber
$x_{3}$	secondary final air flow
$x_{4}$	total gas flow in the SWS chamber
$x_{5}$	total air flow in the SWS chamber
$y_{1}$	$H_{2}S$ concentration (output 1)
$y_{2}$	$SO_{2}$ concentration (output 2)

**Table 2:** Optimal RNN models hyperparameters for the source and target domain Soft Sensors (SSs).

	Delays	Hidden Layers
**Line 2**	Input: 1 Internal: 2 Output: 2	[4,4]
**Line 4**	Input: 4 Internal: 5 Output: 1	[3,3]

**Table 3:** Optimal LSTM models hyperparameters.

	# LSTM Units	# FCL Units	Dropout Prob.
**Line 2**	200	80	0.7
**Line 4**	175	100	0.5

**Table 4:** Optimal RNN and LSTM models for the SRU line 2 performance on the test dataset.

Line 2	RNN		LSTM	
	Output 1	Output 2	Output 1	Output 2
**CC**	0.84	0.84	0.72	0.72
**MAE**	0.13	0.027	0.12	0.16
**RMSE**	0.62	0.67	0.77	0.89

**Table 5:** Optimal RNN and LSTM models for the SRU line 4 performance on the test dataset.

Line 4	RNN		LSTM	
	Output 1	Output 2	Output 1	Output 2
**CC**	0.90	0.91	0.77	0.85
**MAE**	0.02	0.03	0.1 0	0.01
**RMSE**	0.56	0.44	0.70	0.53

**Table 6:** Performances obtained on test data for the SRU line 4 (SSL4) applied to the SRU line 2 dataset. Percentage degradation with respect to the optimal SS for SRU line 2 is reported.

Line 2	RNN		LSTM	
	Output 1	Output 2	Output 1	Output 2
**CC**	0.65 (−22.62%)	0.70 (−16.6%)	0.45 (−37.50%)	0.64 (−11.11%)

**Table 7:** Errors obtained on test data for the SS optimized for the SRU line 4 and applied to the SRU line 2 dataset.

Line 2	RNN		LSTM	
	Output 1	Output 2	Output 1	Output 2
**MAE**	1.11	0.63	1.36	0.62
**RMSE**	1.50	1.17	1.81	1.17

**Table 8:** Model transferred through fine-tuned transferred model (FTTM), performance on test dataset. Degradation in percentage with respect of the optimized models is reported.

Line 2	RNN		LSTM	
	Output 1	Output 2	Output 1	Output 2
**CC**	0.75 (−10.71%)	0.78 (−7.14%)	0.72 (0%)	0.72 (0%)

**Table 9:** Model transferred through FTTM, errors on test dataset.

Line 2	RNN		LSTM	
	Output 1	Output 2	Output 1	Output 2
**MAE**	0.36	0.07	0.23	0.36
**RMSE**	0.81	0.76	0.78	0.92

**Table 10:** Performance on test data obtained after the FTTM procedure using the SRU line 2 reduced training dataset. Percentage degradation with respect of the optimized models is reported.

Line 2	RNN		LSTM	
	Output 1	Output 2	Output 1	Output 2
**CC**	0.63 (−25%)	0.71 (−15.48%)	0.67 (−6.94%)	0.70 (−4.16%)

**Table 11:** Errors on test data obtained after the FTTM procedure using the SRU line 2 reduced training dataset.

Line 2	RNN		LSTM	
	Output 1	Output 2	Output 1	Output 2
**MAE**	0.40	0.61	0.18	0.17
**RMSE**	0.98	1.07	0.85	0.90

**Table 12:** Transferred models for the SRU line 2, using the transferred hyperparameters model (THM) procedure. CC on test data and percentage degradation with respect to the optimized model performance are reported.

Line 2	RNN		LSTM	
	Output 1	Output 2	Output 1	Output 2
**CC**	0.74 (−11.90%)	0.73 (−13.09%)	0.71 (−1.39%)	0.64 (−11.11%)

**Table 13:** Transferred models for the SRU line 2, using the THM procedure. Estimation errors on the test dataset are reported.

Line 2	RNN		LSTM	
	Output 1	Output 2	Output 1	Output 2
**MAE**	0.09	0.50	0.03	0.78
**RMSE**	0.76	0.96	0.77	1.21

## Data Availability

The data presented in this study are available on request from the corresponding author.
